# The Influence of Photodynamic Antimicrobial Chemotherapy on the Microbiome, Neuroendocrine and Immune System of Crustacean Post Larvae

**DOI:** 10.3390/toxics11010036

**Published:** 2022-12-30

**Authors:** Anas Abdulaziz, Athira Vengalil Pramodh, Vrinda Sukumaran, Devika Raj, Ann Mary Valathuparambil Baby John

**Affiliations:** CSIR-National Institute of Oceanography, Regional Centre Kochi, Cochin 682018, Kerala, India

**Keywords:** photodynamic therapy, curcumin, water-associated pathogen, reactive oxygen, aquaculture, neuroendocrine toxicity

## Abstract

Photodynamic antimicrobial chemotherapy (PACT), employing a combination of light and natural photosensitizer molecules such as curcumin, has been accepted as a safe modality for removing aquatic pathogens which cause diseases such as cholera in humans and vibriosis in aquatic animals. Curcumin and its photodegradation products are generally considered as safe to animals, but the impact of reactive oxygen species (ROS) generated by these products on the growth and survival of organisms at a cellular level has not been studied in detail. The ROS generated by curcumin on photoexcitation using blue light (λ_max_ 405 nm, 10 mW cm^−2^) disinfects more than 80% of free-living *Vibrio* spp. in the rearing water of *Penaeus monodon*. However, it is less effective against *Vibrio* spp. colonized inside *P. monodon* because the carapace of the animal prevents the transmission of more than 70% of light at the 400–450 nm range and thus reduces the formation of ROS. The influence of curcumin and photoexcited curcumin on the microbiome of *P. monodon* were revealed by nanopore sequencing. The photoexcited curcumin induced irregular expression of genes coding the moult-inhibiting hormone (MIH), Crustacean hyperglycaemic hormone (CHH)), prophenoloxidase (ProPO), and crustin, which indicates toxic effects of ROS generated by photoexcited curcumin on the neuroendocrine and immune systems of crustaceans, which could alter their growth and survival in aquaculture settings. The study proposed the cautious use of photodynamic therapy in aquaculture systems, and care must be taken to avoid photoexcitation when animals are experiencing moulting or environmental stress.

## 1. Introduction

Extensive studies have been conducted over the past decade on identifying alternative molecules and strategies to reduce or replace the prophylactic and therapeutic application of antibiotics [1,2,3]. Photodynamic therapy (PDT) is a novel approach and could reduce bacterial pathogens without leaving chances of emerging resistance [4]. Recent review summarized the advancements in PDT, and its application in the treatment of cancer, dental infections, skin infections, disinfecting near-patient surfaces, food processing, sewage treatment, and aquaculture [4]. Photodynamic therapy includes a combination of light and a photosensitizer having unique photoluminescence properties. The photoexcited photosensitizer undergoes a photochemical reaction or intersystem crossing to generate its long-lived triplet excited state (T_1_). The triplet excited state species follows two types of reaction mechanisms (Type 1 and Type 2). In the Type 1 mechanism, an electron may be transferred from an excited photosensitizer (PS) to the water and other molecules in the proximity to produce a superoxide, whereas in the Type 2 mechanism, the excited PS transfers the excess energy directly to molecular oxygen to generate excited state ^1^O_2_ [4]. Therefore, synthesized reactive oxygen species (ROS) can induce toxic effects to the biological molecules such as proteins, lipids, and DNA in the proximity. The chemical pollutants also induce oxidative stress in the aquatic systems, which induces ferroptosis, necroptosis, and organ damage in aquatic animals [5,6]. The efficacy of photodynamic therapy may vary depending on the excitation source, interaction of the photosensitizer with the pathogen, and the photo properties of the excitation source.

Higher concentrations of some synthetic dyes that are used as photosensitizers are toxic to animal cells [7], which restricts their application in antimicrobial chemotherapy. The recent studies proposed porphyrin found in hemes and chlorophyll [8], curcumin of turmeric [9], and aloe emodin of Aloe Vera [10] as natural photosensitizers with lower toxicities for clinical applications. The yellow coloured polyphenolic compound, curcumin, extracted from the rhizome of perennial plant *Curcuma longa* (turmeric), has been used as an additive in food preparations, cosmetics, and ethnopharmacology [11]. The photophysical properties and low toxicity of curcumin and its nanoparticle conjugates are attractive qualities for its application as a natural photosensitizer in photodynamic therapy [12]. Recent studies also proposed curcumin-based photodynamic therapy for controlling water-associated pathogens of humans and aquatic animals [13]. Water-associated diseases had threatened the life of approximately 0.3 million children (under the age of five years) globally in just one year (2015) [14]. Assuring safe drinking water to nearly 840 million people across the globe who are deprived of clean drinking water has become a United Nations sustainable development goal (SDG 6) to be achieved by 2030 [15]. Water-associated diseases also induced severe economic loss to aquaculture, where, in shrimp culture alone, India incurred a loss of 1.02 B US during 2018–2019 [16]. Photodynamic therapy using curcumin as a photosensitizer has been experimented with for killing water-associated pathogens that cause diseases to aquatic animals and the spoilage of seafood [17].

Photodynamic therapy can replace the use of antimicrobials in aquatic systems (e.g., aquaculture and sewage treatment); however, the ecotoxicological effects of photosensitizers, their photoexcited products, and ROS are a major concern [18,19,20,21]. The ecotoxicological impacts of weathered products of photosensitizer nanoparticles are known and are mediated through the release of toxic metal ions [22]. The environmental risks associated with phenothiazinium photosensitizers (Methylene blue, toluidine blue, and dimethylmethylene blue) were found to be lower compared with antifungal agents; based on these results, photodynamic therapy was proposed as a safe tool for controlling plant-pathogenic fungi [18]. The nonspecific interactions of ROS can also induce unintended effects on the host cells in the proximity. In the present study, we wanted to evaluate the influence of the photosensitizer, curcumin, on the microbiome and neuroendocrine and immune systems of *P. monodon.* We also wanted to discuss the antimicrobial activity of curcumin and photoexcited curcumin against *Vibrio* spp. in the rearing tanks of *P. monodon*.

## 2. Materials and Methods

### 2.1. Preparation of Curcumin and the Rearing of Artemia nauplii and P. monodon

The stock solution (10 mM) of curcumin (AVT natural products, India) was freshly prepared in DMSO and sterile water (1:1) for each set of experiments. The curcumin powder was first allowed to dissolve in DMSO completely. Then, sterile water was added and mixed well to obtain a uniformly concentrated curcumin solution. The solution was stored in a dark container to avoid light exposure. Different concentrations of curcumin (1, 10, 20, 30, 40, and 50 µM) were prepared in sea water from fresh stock before each experiment.

The *Artemia* nauplii used in the current study were reared following the method of Sorgeloos et al. [23], which had been optimized in our laboratory for use in an earlier study [24]. All materials used for the rearing of *Artemia* were previously sterilized. Briefly, 0.5 g of *Artemia* cysts were hydrated and kept under suspension in 45 mL of tap water in a 250 mL capacity beaker for 1 h by sparging filter-sterilized air from the bottom. Subsequently, the cysts in the suspension were decapsulated by treating them with an equal volume of sodium hypochlorite (4% *w*/*v*) for 5–7 min. The decapsulated cysts were separated from the solution by filtering them through a sieve (100 µm pore size) and then washed several times with sterile seawater to remove residual chlorine. The washed cysts were resuspended overnight for hatching in 100 mL of sterile sea water in a 250 mL beaker provided with aeration and light. The viable healthy *Artemia* nauplii were further distributed into petri dishes for toxicity studies.

The post larvae (PL) of *P. monodon* used in the study were collected from a commercial hatchery (Regional shrimp hatchery, Azhikode, Kerala) and were produced from one mother. Standard rearing protocol of penaeid shrimp and regular monitoring of water quality were followed in the hatchery [25]. The post larvae at stage 16–17 were transported into the laboratory where they were acclimatized with aged seawater (25 psu) for 24–48 h. The post larvae (20 nos) were distributed in a 1000 mL capacity glass beaker containing 500 mL seawater. Each tank was provided with separate aeration, and the post larvae were fed ad libitum with commercially available pelleted feed. All experiments were completed within 48 h of acclimatization in the laboratory. The water quality (temperature, pH, salinity, and ammonia) of the rearing tanks was monitored regularly following standard protocol, and the levels of pH and ammonium were maintained at 7.5 to 8.0 and <0.1 mgL^−1^, respectively, by exchanging 30–40% of the water daily.

### 2.2. Spectral Properties and Toxicity of Curcumin

The UV–Visible absorption spectra of curcumin and transmittance spectra of shrimp carapace were measured in a UV–Vis Spectrophotometer (Shimadzu UV 1800, Shimadzu, Mumbai, India). The absorption spectra of curcumin (25 µM) before and after photoexcitation for 60 min were measured at equal (10 min) time intervals. After recording the initial absorption spectrum, the curcumin solution was exposed to light (10 mW cm^−2^) from a blue LED source (λ_max_ 405 nm). The light transmittance of the carapace of an adult shrimp was also measured. A piece of a cleaned carapace 30 mm in size was inserted vertically in the cuvette holder of the UV–Vis spectrophotometer and the transmittance of light was measured at a range of 250–800 nm.

Different concentrations (1, 10, 20, 30, 40, and 50 µM) of curcumin and their toxicity towards *Artemia* nauplii and *P. monodon* were tested separately. A control without photoexcitation was also maintained, and all experiments were done in triplicates. *Artemia* nauplii (20 nos) were distributed in a six-well plate and each well was supplemented with different concentrations of curcumin in sterile sea water and exposed to light emitted from an LED light source (λ_max_ 405 nm, 10 mW cm^−2^) for 30 min. The numbers of live *Artemia* nauplii were counted after 24 h of treatment with curcumin and photoexcited curcumin.

The toxicity levels of curcumin and photoexcited curcumin towards the post larvae of *P. monodon* were also tested. Here, the post larvae of *P. monodon* were distributed in experimental tanks as outlined in the above section. Separate tanks were maintained for each concentration of curcumin and photoexcited curcumin. The tanks assigned for photoexcited curcumin were exposed to light emitted from an LED light source (λ_max_ 405 nm) for 15 min. The post larvae of *P. monodon* in all tanks were maintained with an adequate supply of air and food ad libitum, and the numbers for ones that survived after 24 h were counted manually. The temperature of the water in all cases of exposure to light was recorded continuously with a sensor immersed in the water (iButton DS1922l#5).

### 2.3. Abundance of Vibrio *spp.* in P. monodon Exposed to Photoexcited Curcumin

Crustaceans are the natural carriers of *Vibrio* spp., and they succumb to disease outbreaks caused by these bacteria at times depending on water quality deteriorations in the larval rearing systems [3,26]. The abundance of free-living and animal-associated *Vibrio* spp. in the rearing tanks of *P. monodon* exposed to different concentrations of curcumin and light was monitored by a standard culture-dependent method. Samples were collected before and after 1 h of the photoexcitation experiments. Water samples were serially diluted up to six times with saline (0.8% NaCl), and 100 µL from each dilution were spread over the surface of a *Vibrio*-specific medium, TCBS agar. Larval samples were macerated with saline and diluted up to six times. One hundred microlitres of sample from each dilution were spread over the surface of the TCBS agar. All plates were incubated at 37 °C for 24 h and the numbers of colonies per plate were counted. The numbers of colonies in the water sample and in the animal were expressed as colony forming units (cfu) mL^−1^ and cfu g^−1^, respectively. The percentage reduction in the abundance of *Vibrio* spp. in each treatment was calculated in comparison with the microbial cell count in the same sample before the treatment.

### 2.4. Microbiome of P. monodon Exposed to Photoexcited Curcumin

Post larvae exposed to different concentrations of curcumin (0, 10, 30, and 50 µM) in the absence and presence of photoexposure were collected and preserved immediately in RNA*later* solution (Sigma Aldrich, Bangalore, India) and stored at −20 °C freezer for DNA extraction. Total DNA extractions were performed from all these samples with Dneasy Blood & Tissue Kit (Qiagen India Pvt Ltd., Delhi, India). The quality of DNA was checked on an agarose gel, and DNA quantification was done on a nanodrop spectrophotometer. DNA extractions diluted to a uniform concentration of 50 ng/µL were used as the template for PCR amplification along universal full length 16s rRNA Primers tailed with a PCR adaptor sequence and a LongAmp Taq 2× master mix (New England Biolabs, MA, USA). The amplicons obtained were purified using 1.6× AMPure XP beads (Beckmann Coulter India Pvt Ltd., Bangaluru, India). Precisely 25 ng of purified amplicons were barcoded using a second PCR reaction with the LongAmp Taq 2× (NEB, USA) and then cleaned up again with 1.6× AMPure beads. The barcoded amplicons were quantified using Qubit and were pooled into one at equimolar concentration. Then, end repair of this sample was performed using a NEB next ultra II end repair kit (NEB, USA) and further purified with 1× AMPure beads. Next, Adapter ligation (AMX) was performed with the sample using a NEB blunt/TA ligase (NEB USA) for 15 min. The final Library mix was cleaned up again using AMPure beads and finally eluted into 15 μL of elution buffer. Nanopore Sequencing was performed on an Mk1C device (Oxford Nanopore Technologies, Oxford, UK) using SpotON flow cell R9.4 (FLO-MIN106) in a 48 h sequencing protocol.

The long-read raw data obtained from a Nanopore sequencer were base-called and de-multiplexed using Guppy v2.3.4 available on the ONT community site (https://community.nanoporetech.com, accessed on 28 September 2022). The barcodes and adapters from the reads were removed using Porechop (https://github.com/rrwick/Porechop, accessed on 30 September 2022). The sequences were filtered with NanoFilt [27] to remove reads with a quality score smaller than 9 and a read length between 100 bp and 1800 bp. Read quality statistics and plots were obtained using NanoPlot [27] and EPI2ME Desktop Agent software provided by Oxford Nanopore Technologies (ONT). A quantitative assessment of taxonomic representation within each sample was done by a wf–metagenomic Nextflow workflow in EPI2ME Labs provided by ONT using the NCBI 16S + 18S rRNA database. This workflow uses Kraken2 [28] and minimap2 [29] programs for the taxonomic classification of sequences from metagenome samples. Alpha and Beta diversity matrices of the samples were obtained using QIIME2 pipeline version 2022.8 [30]

### 2.5. Neuroendocrine (MIH, CHH) and Immune (Crustin, PoPO) Gene Expression

Post larval samples were collected before treatment and then one and two days after treatment with curcumin and photoexcited curcumin; the samples were stored in RNA*later* solution (Sigma, St. Louis, MO, USA) at −20 °C for RNA extraction and further analysis of gene expression. The total RNA was extracted from tissue samples of the animal using commercially available Trizol (Thermo Fisher Scientific Inc, Waltham, MA, USA). The genomic DNA was removed by treating the total RNA with Rnase-free Dnase (0.2 U) for 10 min at 37 °C and at 75 °C for 10 min. The purity and concentration of this extracted RNA were confirmed by measuring the absorbance (Abs 260/280 nm) in a nanodrop (ND1000 spectrophotometer V3.8). An aliquot of 1 µg total RNA was reverse-transcribed in a 20 µL reaction volume using Monster kit first strand cDNA synthesis kit (Epicentre Biotechnologies, Madison, WI, USA) following the instruction protocol.

Expression of neuroendocrine hormones (moult-inhibiting hormone (MIH) and Crustacean hyperglycaemic hormone (CHH)), and immune- (Prophenoloxidase (ProPO) and antimicrobial peptide- (Crustin) genes was measured by an absolute quantitative real-time PCR (qRT-PCR) method. Details on the preparation of standards for the RT-PCR and their confirmation using cloning and sequencing were described in our previous manuscript [31]. Primer sets used for the PCR were given in Table 1. The PCR amplification was performed in a total volume of 10 μL containing 2 × SYBR Premix Ex Taq II (Tli Rnase H Plus) (Takara, California, USA), 1 μL of cDNA (1/25 diluted), and 10 pm/μL of each primer. The qRT-PCR program was 95 °C for 600 s followed by 45 cycles of 95 °C for 10 s, 56 °C for 10 s and 72 °C for 10 s; completion was with a dissociation (melt curve) analysis of amplified products performed at the end of each cycle to confirm the amplification and detection of confirmed PCR product. cDNA of the post larvae of *P. monodon* collected prior to and after treatment with curcumin and photoexcited curcumin were used for analysis of MIH I, CHH, ProPO, and crustin. Plasmids containing MIH (119 bp), CHH (113 bp), crustin (119 bp), and ProPO (122 bp) were used to make 10-fold dilutions from 1.88 × 10^9^ (MIH I), 2.59 × 10^9^ (CHH), 1.31 × 10^10^ (crustin), and 5.21 × 10^8^ (ProPO) copies down to one copy of the starting molecule. The obtained plasmid DNA standard curve equations were used to calculate the absolute copy number of three genes in the test and control samples. Each sample was run in triplicates for each gene and each day of the experiment. The absolute quantification data obtained were analysed using Roche Lightcycler 96 software (Roche, San Francisco, CA, USA).

### 2.6. Statistical Analysis

The statistical significance of the differences obtained between the curcumin and the photoexcited-curcumin-treated groups on the toxicity and expression of neuroendocrine and immune genes was tested by applying a single factor ANOVA (*p* < 0.05). All experiments were conducted in triplicates and the values are presented with ± standard deviation (sd).

## 3. Results

### 3.1. Spectral Properties and Toxicity of Curcumin

**Figure 1:**
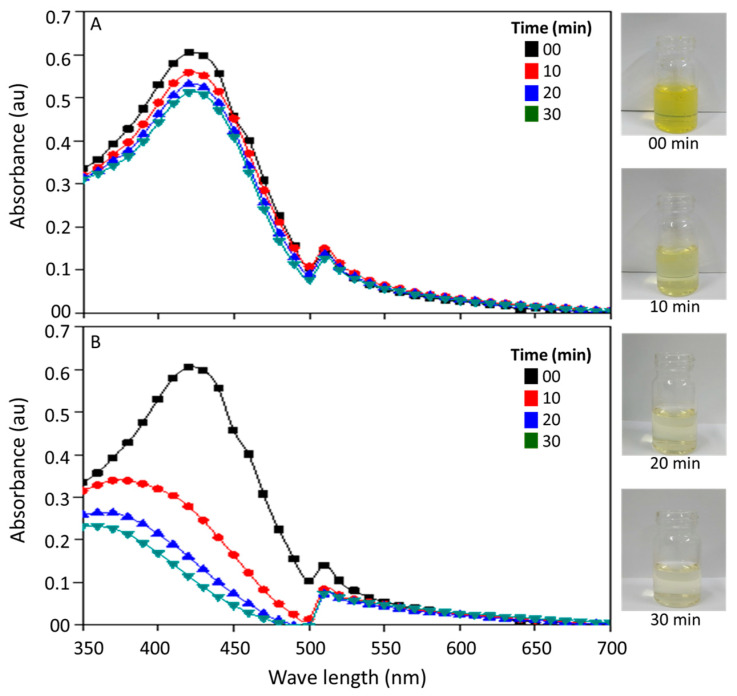
Changes in the absorption spectra of curcumin at different time intervals on exposure to dark (**A**) and light (**B**). The images of curcumin solution at different time intervals of photoexcitation are also given on the right side.

Curcumin had a broad absorption in the violet and blue (380–500 nm) region of light with a peak at approximately 420 nm under dark conditions (Figure 1A). This indicates the possibility of exciting curcumin with a light source having an emission peak at approximately 400–450 nm. The light exposure during the photodynamic therapy reduced the colour and absorption properties of curcumin in water (Figure 1B). The absorption spectra of curcumin remained intact under dark condition (Figure 1A) while they showed a shift towards the violet region with a reduction in absorption peak with increasing exposure to light from an LED source (Figure 1B). This was also evident in the images of the curcumin solution, which became apparently clear after 30 min exposure to light.

**Figure 2:**
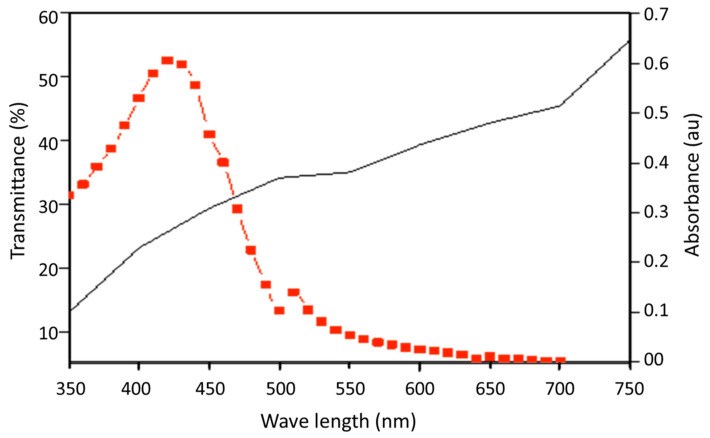
Light transmittance (%) of shrimp carapace (black) and absorbance spectrum (red) of curcumin.

**Figure 3:**
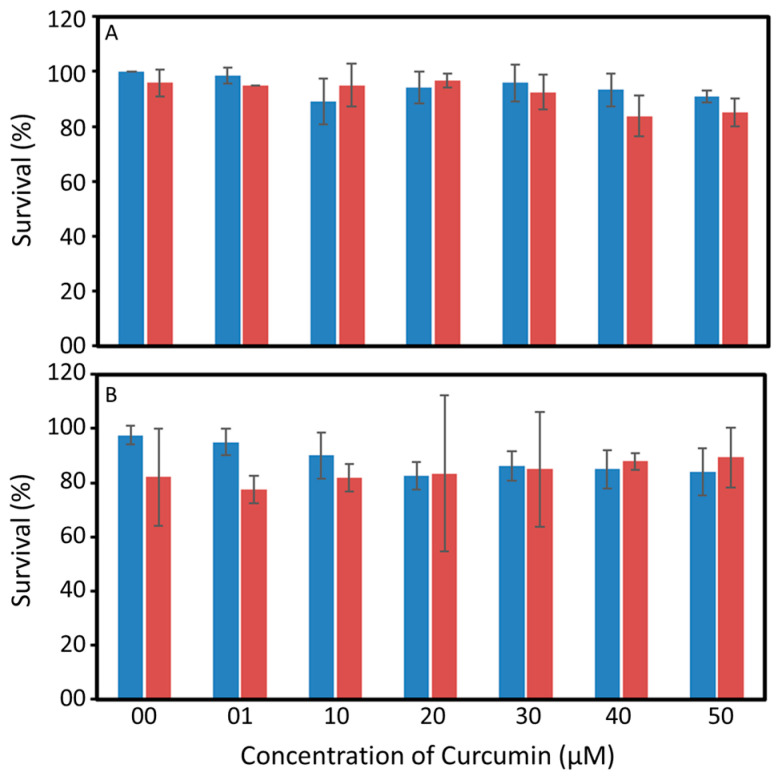
Toxicity of Curcumin (blue) and photoexcited curcumin (red) to *Artemia* nauplii (**A**) and post larvae of *Penaeus monodon* (**B**). Results are presented as average ± standard deviation (sd). The differences obtained in the toxicity were not statistically significant (*p* > 0.05).

Although the blue light can pass through the water and excite curcumin, the shell (i.e., carapace) of certain aquatic animals (e.g., Crustaceans) blocks the transmission of blue light (Figure 2). The carapace of *P. monodon* transmitted less than 30% of the light in the desired wavelength of <450 nm (Figure 2). The curcumin was not toxic to the *Artemia* nauplii or the post larvae of *P. monodon* (Figure 3). *Artemia* nauplii exposed to 50 µM curcumin and photoexcited curcumin for 24 h showed a survival rate of 90.8 ± 2% and 85 ± 5%, respectively. The survival rate of *P. monodon* was 97.5 ± 3. 5 % and 83.8 ± 8.6 % at 24 h after exposure to zero and 50 µM curcumin, respectively, and 82.1 ± 17.9% and 89.3 ± 11.1% for photoexcited curcumin under the same conditions.

### 3.2. Abundance of Vibrio *spp.* and Microbiome of Shrimp Larvae Exposed to Photoexcited Curcumin

**Figure 4:**
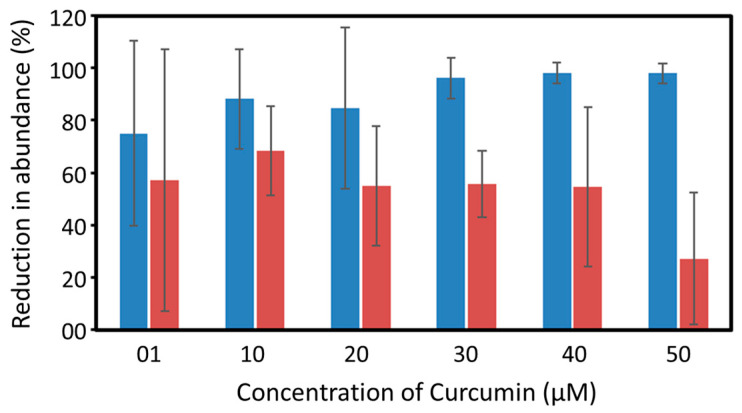
Reduction in the abundance of viable *Vibrio* spp. colonies in water (blue) and post larvae of *P. monodon* (red) on exposure to different concentrations of photoexcited curcumin. Results are presented as average ± standard deviation (sd).

*Vibrio* spp. are the natural inhabitants of the larval rearing systems and are found as free-living organisms or in association with the larvae and live-feed of the larvae [3,26,33]. The abundance of the residential population of *Vibrio* spp. in the water column and the animal samples of the rearing tank was in the order of 10^3^ cfu mL^−1^ and 10^6^ cfu g^−1^, respectively. Our results indicated that more than 80% of the residential population of *Vibrio* spp. in the water column were disinfected on exposure to 10 µM of photoexcited curcumin and an almost complete reduction was observed at 30 µM (Figure 4). A similar response was not found among the post larvae of *P. monodon* on exposure to photoexcited curcumin (Figure 4). A maximum of 68 ± 17% reduction in the abundance of *Vibrio* spp. associated with the animal samples was recorded on exposure to 10 µM of photoexcited curcumin, which remained static up to 40 µM. A notable reduction in the abundance of *Vibrio* spp. associated with *P. monodon* was observed on exposure to 50 µM of photoexcited curcumin.

Similar results were also observed on the microbiome composition of *P. monodon* exposed to curcumin and photoexcited curcumin. The abundance of phylum proteobacteria, under which *Vibrio* spp. are listed, reduced with exposure to increasing concentrations of photoexcited curcumin (Table 2). The changes in the values of diversity indices also confirmed the influence of different concentrations of curcumin and photoexcited curcumin on the microbiome composition of *P. monodon*. The reduced abundance of firmicutes, actinobacteria, and Bacteroidetes also confirms the broad-spectrum activity of ROS.

### 3.3. Neuroendocrine and Immune Gene Expression

**Figure 5:**
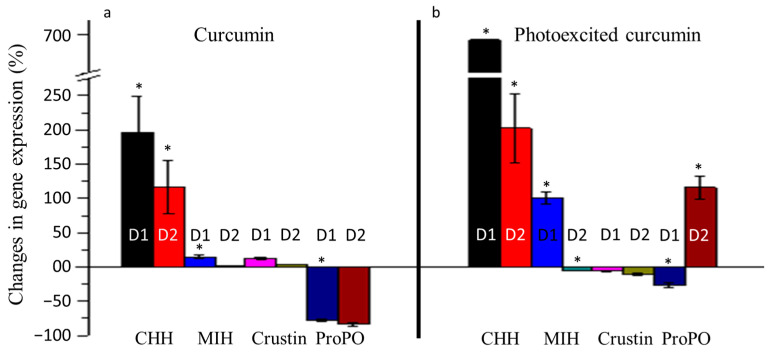
Changes in the expression levels of neuroendocrine (CHH, MIH) and immune (Crustin and ProPO) genes of *Penaeus monodon* larvae on exposure to curcumin and photoexcited curcumin. Samples were analysed one (D1) and two (D2) days after exposure to curcumin (**a**) and photoexcited curcumin (**b**). The differences observed between both treatment groups are statistically significant (marked as *, *p* < 0.05).

We studied the changes in the expression levels of neuroendocrine and immune genes in the post larvae of *P. monodon* on the 1st and 2nd day after exposure to curcumin and photoexcited curcumin (Figure 5). The level of expression on the 1st and 2nd day after the treatment was calculated in comparison with that of 0 day (i.e., before exposure to curcumin or photoexcited curcumin). On the 1st day, the expression of the CHH gene increased by 196 and 692 times, respectively, in *P. monodon* exposed to curcumin and photoexcited curcumin. The expression of the MIH gene also increased by 15 and 101 times, respectively, in animals exposed to curcumin and photoexcited curcumin on the 1st day. The level of expression of the CHH and MIH genes reduced on the 2nd day after treatment. The immune genes showed irregular expression on treatment with curcumin and photoexcited curcumin. An increase by 13 and 3 times in the expression of crustin was observed on the 1st and 2nd day, respectively, after exposure to curcumin, while there was a decrease in those groups exposed to photoexcited curcumin. A decrease by 78 times in the expression of ProPO was observed in the post larvae of *P. monodon* on the 1st and 2nd day after exposure to curcumin alone. The expression of ProPO in *P. monodon* decreased by 27 times on the 1st day and increased by 116 times on the 2nd day after exposure to photoexcited curcumin.

## 4. Discussion

Many studies have shown the efficiency of PACT in controlling bacterial infections, and their application in preventing pathogens has become the basis of clean water and sanitation under the United Nations sustainable development goal (SDG 6) [4,13]. Curcumin, the polyphenolic pigment extracted from the rhizome of *Curcuma longa*, has been accepted as a natural photosensitizer in photodynamic therapy to disinfect water-associated, food-borne, and cutaneous pathogens [13,34]. Curcumin and its photoexcited products are not toxic to animals, and clinical studies have shown that daily consumption of 12 g of curcumin for three months is safe for animals [35]. The broad absorption spectrum of curcumin varies in organic and aqueous solvents depending on the solvent characteristics, temperature, and substitutions of functional groups in the curcumin [13]. The changes in the absorption properties of photoexcited curcumin are due to the self-sensitized pseudo-unimolecular degradation by electron transfer from the excited state to molecular oxygen in the solution [13,36,37]. Several photoactive intermediates and products are generated during the photosensitized oxygenation and ROS-mediated degradation of curcumin [13,37].

Curcumin generates singlet oxygen on photoexcitation, which rapidly disinfects the planktonic bacteria in the local environment through disrupting their cell wall [13]. The excited-state properties of curcumin, such as the triplet energy (ca. 191 kJ mol^−1^), triplet quantum yield (ca. 0.11), and the triplet lifetime (ca. 1.5 µs), support their abilities to generate singlet oxygen, while the initiation of ROS pathways through electron transfer to oxygen and superoxide generation are possibilities. Our previous experiments using molecular sensors (9,10-anthracenediyl-bis(methylene) dimalonic acid (ADMA) and N-[10-(9-,10-dimethylanthracen-2-yl)-7-(dimethylamino)-5,5-dimethylbenzo(b,e)siliN-3(5H)-ylidene]-N-methylmethanaminium chloride (SiDMA) also confirmed the abilities of curcumin to produce singlet oxygen on photoexcitation [13]. ROS generated by photoexcited curcumin have been shown to be efficient in disinfecting bacterial pathogens associated with oral infections [38,39], spoilage of food [40,41], water-associated pathogens [13], and biofilm-forming bacteria associated with plastics [9]. The alterations in the microbiome of penaeid shrimps in response to elevated ROS have been reported under various clinical conditions and heavy metal stresses [42,43]. First generation photosensitizers such as rose Bengal and toluidine blue were shown to kill both Gram-negative and -positive bacteria by disrupting their cell wall [4,24,44]. However, the ROS-mediated killing of pathogens is largely restricted to the immediate proximity of a photosensitizer due to the short lifetime of singlet oxygen [45]. Several strategies, including the biofunctionalization of photosensitizers to encourage their site-specific delivery [46], increasing the concentration of ROS through supply of oxygen by conjugating with a photosynthetic organism [47], enhancing the coverage area of ROS through enzymatic micromotors [48], and increasing lifetime by using enhancers of ROS such as potassium iodide, have been experimented with in the recent past to overcome the limitation of the short lifetime of ROS in photodynamic therapy.

The reduced activity of photoexcited curcumin against *Vibrio* spp. associated with post larvae of *P. monodon* compared with that of water could be attributed to the lower chances of ROS generation within the animal body due to a low penetration of light, as evident from the spectral properties of the carapace of *P. monodon* and the innate antioxidant mechanisms of the animal. The carapace prevented the transmission of over 70% of the light at this wavelength (400–450 nm), which could reduce the efficacy of ROS generation. The chitinous exoskeletons of crustaceans are the primary attachment point of *Vibrio* spp., wherein they are protected from chemical disinfectants [49,50]. Aquatic crustaceans such as copepods and shrimps are known as the reservoirs of pathogenic *Vibrio* spp. [26]. This has created a major hurdle in eradicating *V. cholerae* from drinking-water sources and pathogenic *Vibrio* spp. in aquaculture settings. A 68 ± 17% reduction in *Vibrio* spp. abundance is promising as it could reduce the chances of disease outbreaks. Nanopore sequencing results of the microbiome of *P. monodon* also confirmed that the relative abundance of proteobacteria, firmicutes, and actinobacteria were decreased with exposure to increasing concentrations of photoexcited curcumin. Results indicate the potential of this technique for reducing the load of pathogenic *Vibrio* spp. in aquaculture systems. Alterations in the microbiome of penaeid shrimps were observed on exposure to metal ions, free radicals, microplastics, and toxic chemicals [43,51,52,53]. The remarkable point is that the reduction in pathogen load is achieved without using any toxic chemicals or the emergence of resistance.

Although curcumin and its photodegradative products are safe for disinfecting water-associated pathogens, their impact on the cellular mechanisms affecting the growth and survival of other organisms living in the same water column are still debatable because the ROS generated during therapy are nonspecific. The current study reported the changes in the expression levels of neuroendocrine and immune genes of *P. monodon* exposed to curcumin and photoexcited curcumin. The higher expression of the MIH I gene by seven times in those groups exposed to photoexcited curcumin compared with curcumin indicates the role of ROS in the over-expression of neuroendocrine genes. The over-expression of MIHs, clustered mostly in the eyestalk of *P. monodon*, may inhibit the synthesis of ecdysteroid in Y-organs and that could alter the moulting frequency and growth of animals [54,55]. Similar variations in the expression of MIH I genes were reported from crustaceans exposed to irregular light/dark cycles, and was induced by ROS stress which led to alterations in the circadian rhythm and arrhythmic moulting [56]. The CHH gene expression controls the glucose metabolism and involves in maintenance of general physiology in crustaceans [57]. The over-expression of the CHH gene induced in photoexcited curcumin treatment may contribute to the induction of phagocytosis and stimulation of immune functions in *P. monodon.* Over-expression of the CHH gene and hyperglycaemia are a general response of many aquatic animals towards environmental stress, metal pollution, and infections [58,59,60]. The ROS accumulation created by the photoexcited curcumin also would have triggered the expression of CHH in the same way a bacterial infection does. High concentrations of ROS act as major effectors of the ProPO system; the slow build-up of ROS by the photoexcited curcumin reversed the initial downregulation of ProPO on the second day of treatment. An enhanced immune response toward curcumin has been reported in shrimps and fishes [61,62,63], but a weakening of the immune system for a short time by the photodegradation products of curcumin has been reported for the first time.

## 5. Conclusions

Results of the study recommend photodynamic antimicrobial chemotherapy with curcumin as a photosensitizer as a propitious strategy to disinfect water-associated pathogens in the larval rearing system of *P. monodon* while the implications of photoexcited curcumin on the animal is cautioned. Curcumin and photoexcited curcumin can cause changes in the expression levels of moult-inhibiting hormone (MIH) and Crustacean hyperglycaemic hormone (CHH), which may impinge the animals’ growth and survival. Therefore, photodynamic therapy should be avoided in aquaculture systems when animals are undergoing moulting or are exposed to environmental stress. The duration of photoexcitation should be kept to a minimum to avoid the build-up of ROS beyond the tolerance limit of the organisms being cultivated.

## Figures and Tables

**Table 1:** Primer details of three genes used for absolute quantitative real-time PCR analysis.

Sl No.	Gene	Primer Sequence (5′-3′)	Tm (°C)	Amplicon Size (bp)	Reference
1.	MIH	F- TAGTGCGTGTGTGTGAGGAT R- CCTGTTGGCAGCCTTTAGAC	56	119	[31]
2.	CHH	F- GCCGAATGCAGGAGTAACTG R- TTGCCGAGCCTCTGTAGG	56	113	[31]
3.	Crustin	F- AGTTCCTGGAGTTGGAGGTGGATT R- ACCTCGTTCTGCAGTAATTGCACTC	56	119	[32]
4	ProPO	F- CGGTGACAAAGTTCCTCTTC R- GCAGGTCGCCGTAGTAAG	56	122	[32]

**Table 2:** Microbiome analysis of *P. monodon* exposed to different concentrations of curcumin in the presence and absence of photoexcitation.

	Without Photoexcitation				With Photoexcitation			
Curcumin (µM)	0	10	30	50	0	10	30	50
Total read of nanopore sequencing	671,518	119,439	145,340	158,835	366,319	330,755	178,973	169,091
Reads filtered (>300 bp)	439,981	84,764	98,935	95,529	249,984	222,984	139,451	131,852
Diversity indices								
Chao 1	174,075.9	50,176.9	74,701.9	74,791.1	120,947.1	136,122.7	115,726.3	116,070.5
Shannon	13.78	10.26	12.97	12.15	12.98	12.17	9.65	10.42
Top 5 abundant bacterial phylum (%)								
Proteobacteria	15.39	8.26	9.87	13.43	14.42	9.13	2.5	3.7
Firmicutes	1.75	5.26	2.42	1.51	2.48	1.82	0.71	0.17
Actinobacteria	0.64	0.47	0.38	0.25	0.89	0.26	0.11	0.03
Bacteroidetes	0.64	0.11	0.44	0.41	0.57	0.39	0.14	0.19
Thermotogae	0.10	0.20	0.30	0.27	0.49	0.35	00	00

## Data Availability

The Nanopore metagenome sequences were deposited to DDBJ/ENA/GenBank under BioProject accession number PRJNA916655, BioSample accession numbers SAMN32489654, SAMN32489655, SAMN32489656, SAMN32489657, SAMN32489658, SAMN32489659, SAMN32489660, SAMN32489661 and SRA accession numbers SRR22924198, SRR22924199, SRR22924200, SRR22924201, SRR22924202, SRR22924203, SRR22924204.
